# The Effect of Adoption of an Electronic Health Record on Duplicate Testing

**DOI:** 10.1155/2016/1950191

**Published:** 2016-03-21

**Authors:** Todd C. Kerwin, Harmony Leighton, Kunal Buch, Azriel Avezbadalov, Hormoz Kianfar

**Affiliations:** New York Hospital Medical Center of Queens/Weill Medical College of Cornell University, Flushing, NY 11355, USA

## Abstract

*Background*. The electronic health record (EHR) has been promoted as a tool to improve quality of patient care, reduce costs, and improve efficiency. There is little data to confirm that the use of EHR has reduced duplicate testing. We sought to evaluate the rate of performance of repeat transthoracic echocardiograms before and after the adoption of EHR.* Methods*. We retrospectively examined the rates of repeat echocardiograms performed before and after the implementation of an EHR system.* Results*. The baseline rate of repeat testing before EHR was 4.6% at six months and 7.6% at twelve months. In the first year following implementation of EHR, 6.6% of patients underwent a repeat study within 6 months, and 12.9% within twelve months. In the most recent year of EHR usage, 5.7% of patients underwent repeat echocardiography at six months and 11.9% within twelve months. All rates of duplicate testing were significantly higher than their respective pre-EHR rates (*p* < 0.01 for all).* Conclusion*. Our study failed to demonstrate a reduction in the rate of duplicate echocardiography testing after the implementation of an EHR system. We feel that this data, combined with other recent analyses, should promote a more rigorous assessment of the initial claims of the benefits associated with EHR implementation.

## 1. Introduction

The electronic health record (EHR) has been promoted as a tool to improve quality of patient care, reduce costs, and improve efficiency. In 2005, the RAND Corporation released a report estimating that the widespread implementation of EHR could save the healthcare system over $80 billion annually [1]. The Health Information Technology for Economic and Clinical Health (HITECH) Act, part of the American Recovery and Reinvestment Act of 2009, was enacted to “promote the adoption and meaningful use of health information technology.” Under the HITECH Act, the federal government committed to spending $25.9 billion to increase the implementation of health information technology [2]. Title IV of the act provides for incentive payments to hospitals and providers that adopt a certified EHR beginning in 2011. Those that do not adopt a certified EHR by 2015 will be penalized 1% of Medicare payments, increasing to 3% over 3 years.

Amongst other benefits, implementation of EHR was predicted to reduce the ordering of duplicate testing. Duplicate testing appears to be prevalent in current medical practice. A recent study reported that 55% of Medicare beneficiaries underwent a repeat echocardiogram within three years [3]. By providing practitioners with easy access to prior results, the EHR could be expected to reduce the ordering of a redundant test. Additionally, the accessibility of the entire medical record will give the practitioner further patient data which should improve their clinical decision making process. Early data, performed under highly controlled circumstances, as well as predictive models validated this hypothesis [1, 4–6]. However, little data exist to confirm this in a real-world setting. Frequently, the EHR system is selected by administrators with less than optimal buy-in by clinicians and used in an out-of-the-box fashion with minimal customization. Therefore it is crucial to examine the performance of EHR as it actually being implemented. Recent research found that, in an outpatient setting, diagnostic test ordering actually increased by 40–70% when practitioners had access to computerized records [7]. We sought to examine the effect of the adoption of EHR on the ordering of duplicate transthoracic echocardiography in a hospital-based laboratory.

## 2. Methods

### 2.1. Design and Setting

This is a single-site, retrospective study performed at a 500-bed urban teaching hospital. Since 2005, echocardiographic studies have been reported electronically using the Medcon system (McKesson, San Francisco, CA). A desktop version is available on all computer terminals throughout the hospital for both report retrieval and image review. In January 2009, our institution phased in the Sunrise Clinical Manager (Allscripts, Chicago, IL). The system incorporates clinical documentation, order entry, pharmacy, radiology, and laboratory components for all inpatient and Emergency Department services. Cardiac imaging, such as echocardiography and invasive cardiology, studies remained on a separate system as the two software applications were not interfaced. The results and images however could be reviewed on the same computer station as the Allscripts EHR.

### 2.2. Data Collection and Statistical Analysis

In order to assess the rate of duplicate test ordering, we reviewed all inpatient and outpatient echocardiograms performed at our laboratory for a twelve-month period prior to institution of the EHR (from July 2007 to June 2008). We then assessed whether an echocardiogram was repeated within six or twelve months of the performance of the index study. A duplicate test was defined as the performance of another full echocardiogram within six or twelve months of the index study. Echocardiograms which were ordered as a focused exam or for reassessment of a specific prior finding were not included as duplicate studies. We compared this pre-EHR rate of duplicate test ordering to that of the twelve-month period after implementation of our EHR (July 2009 to June 2010) and to the most recent year of EHR usage (July 2011 to June 2012). The July to June period was chosen to correlate with the academic year of the postgraduate trainees, who enter the majority of the patient orders at our institution. Demographics including age and gender were recorded. Additionally the indication for ordering of the test was recorded. The rate of duplicate test performance before and after EHR implementation was compared using a *χ* ^2^ analysis.

## 3. Results

**Figure 1:**
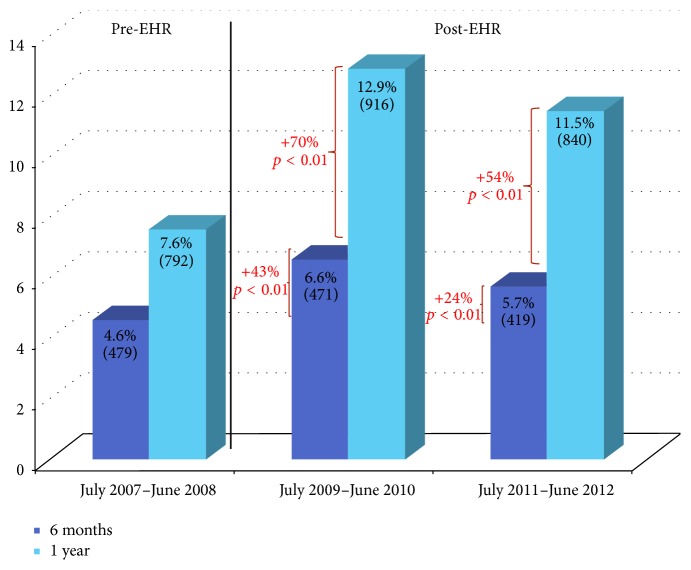
Rate of duplicate echocardiography testing before and after the implementation of the electronic health record. Baseline rate of duplicate echocardiography at 6 months and 1 year after the index study prior to implementation of the electronic health record (EHR) is compared to the rates of duplicate testing in year 1 and year 2 after the implementation of the electronic health record (*p* < 0.01 for all comparisons of pre-EHR to post-EHR).

In the twelve-month period prior to the implementation of EHR 10,399 patients underwent a transthoracic echocardiogram. Within six months of the index exam, 4.6% of patients underwent a full repeat study, and within twelve months 7.6% of patients underwent duplicate testing. In the first year following implementation of the EHR, a total of 7,103 patients underwent transthoracic echocardiography. Within six months of the index exam, 6.6% of patients underwent a repeat study, representing a 43% increase compared to the pre-EHR rate (*p* < 0.01). Within twelve months, 12.9% of patients underwent a repeat echocardiogram, representing a 69% increase compared to the pre-EHR period (*p* < 0.01) (Figure 1).

The trend of a higher rate of repeat testing continued in the most recent year of EHR usage. In the second post-EHR year that we examined, a total of 7,317 patients underwent a transthoracic echocardiogram. Within six months of the index study, 5.7% of patients underwent another full echocardiogram, representing a 24% increase in duplicate testing when compared to the pre-EHR rate (*p* < 0.01). Within twelve months, 11.9% of patients were retested, with a 51% increase compared to pre-EHR (*p* < 0.01). However, there did appear to be a decrease in the rate of duplicate testing when comparing the most recent year to the first year of EHR usage. There was an 11% decrease in the rate of duplicate testing at 12 months between these two time periods (*p* < 0.01) (Figure 1).

The demographic characteristics and indications for the study were also assessed. The gender and age distribution remained constant over time, with the exception of a slight decrease in mean age seen in the most recent period. Study indications remained similar over time (Table 1).

## 4. Discussion

We found that, in each of the two twelve-month periods we examined following implementation of EHR at our institution, there was not a decrease in ordering of repeat transthoracic echocardiograms. Rather, we observed a significant increase in the rate of duplicate testing. We did observe a reduction in duplicate ordering between the first year of EHR usage and the most recent year.

We hypothesize several explanations for our findings. The order entry portion of our EHR contains several order sets with an option to order a transthoracic echo. It is possible that this suggests to the practitioner that the study is deemed recommended for that admission diagnosis. After the first year of EHR usage, we entered a statement into the order describing common clinical scenarios when the ordering of an echocardiogram might be appropriate. This might help to explain part of the improvement in duplicate testing rate between the first and most recent EHR years.

In addition to this “power of suggestion” hypothesis, it is possible that the ease of entering an electronic order by clicking one box was more efficient and therefore more attractive than looking in the medical record to review the previous study. We call this the “path of least resistance” hypothesis.

We recognize several limitations to our study. Our study did not address whether the duplicate study was appropriate according to published criteria [8]. It was beyond the scope of our ability to review the medical records of this large sample of patients and score based on appropriateness criteria. Previous data suggests that repeat echocardiograms are more often inappropriate than first-time studies [9]. We did exclude repeat studies ordered to examine specific findings, such as assessing pericardial effusion or left ventricular systolic function after an event. Certainly some of the repeat studies would be deemed appropriate, but it is not clear why the rate of duplicate testing would change significantly over time.

Although we observed an increase in the rate of duplicate testing after implementation of EHR, it is possible that other unquantified confounders contributed to this increase. We found that gender, age, and study indications remained similar over the course of the study; however it is possible that other patient factors changed over time.

Additionally we observed a decrease in the total volume of echocardiograms performed at our institution. The reasons for this are not entirely clear. Our hospital did experience a similar decrease in the volume of services performed in the other cardiovascular laboratories. For comparison purposes, during the time frame of our study (2007–2012), there was a 28.3% decline in the volume of procedures performed in the cardiac catheterization laboratory. The decrease in monthly volume began 6 months prior to EHR implementation and was likely related to a change in referral patterns which occurred in mid-2008. There is no identifiable link between this change in overall volume and the need for duplicate testing; however this remains as a potential confounder of our observation. There are approximately 1,300 attending physicians who have privileges at our institution, therefore controlling for the ordering patterns of these doctors was beyond our means. The overwhelming majority of these physicians are voluntary (not employed by the institution) and therefore stand to reap no financial gain by ordering echocardiograms on hospitalized patients.

Perhaps, most importantly, the electronic systems containing the echocardiography reports and order entry were not interfaced. The interfacing of electronic record systems made by different vendors remains a challenge faced by many institutions when their EHR systems are installed out of the box. Many of the advantages of EHR are predicated on the fact that these systems are fully integrated; however, in a real-world environment, as exemplified by our study, this cannot necessarily be accomplished. In our case, budgetary constraints precluded the project of integrating these systems. Unfortunately, this is likely the case at many practice locations which were incentivized by the government to install an EHR. Full optimization and integration, as occurred in the studies which justified the benefits of EHR, likely have not taken place at many sites.

The potential for improvement in care and cost savings provided the impetus for the federal government to incentive hospitals and practices to rapidly adopt a certified EHR. In the period since the 2005 RAND study was published, annual health care spending has increased by $800 billion [10]. Several recent analyses have found unintended consequences of health information technology as it is implemented in the real world.

A computerized clinical documentation system provides the ability to rapidly populate a medical note. There is a concern that this more efficient means of generating what appears to be a very thorough note has led to upcoding of medical bills. From 2001 to 2010 there has been a shift in billing of patient encounters from the lowest to the highest billing codes. A 2012 report performed by the Department of Health and Human Services Office of the Inspector General found a 17% increase in the usage of the highest two codes for outpatient office visits and a 21% increase in the usage of the top level 5 billing code for emergency room visits [11]. While it is not clear whether EHR has contributed to this drift to higher billing codes it has been observed that institutions which have received incentive payments for adoption of a certified EHR have seen a 47% rise in Medicare payments, compared to a 32% rise at hospitals that have not adopted EHR [12]. The federal government has taken notice of institutions that disproportionately bill higher codes and has encouraged its billing contractors to examine billing patterns [11]. Some CMS billing contractors have warned doctors that it would not reimburse services substantiated by copied documentation [12].

Medical documentation is an essential component of quality care. An accurate medical record is necessary for a multidisciplinary team to effectively care for a patient. A study comparing EHR to paper medical record in 47 community-based practices found that the frequency of documentation of health history and preventive service indicator items was no better in those using an EHR [13]. A recent study performed in the Intensive Care Unit of a large teaching hospital found that 74% of attending physician notes contained information copied from a previous note [14]. On average 61% of the information contained in the note was copied. The phenomenon of “note bloat” whereby excessive and extraneous copied material creates a voluminous daily progress note is now well described [15, 16]. However, clear documentation of an analytical impression and plan is frequently lacking.

In regard to the ability of EHR to reduce the rate of diagnostic testing, results have been inconsistent. Early data seemed to predict that a computerized record could reduce duplicate testing [3, 4]. Recently, a study of over 28,000 outpatient office visits analyzed the effect of access to computerized records on the rate of ordering of imaging studies. A multivariate analysis showed that physicians with access to computerized records were 44% more likely to order an imaging study [1].

A recent commentary by Kellermann and Jones acknowledges that the implementation of EHR has not yet fulfilled its potential [10]. The authors outline several reasons for this, including a slow rate of adoption, lack of interconnectivity of systems, difficulty to use systems, and importantly the persistence of a healthcare system that incentivizes volume rather than value. It is likely that interconnectivity of our cardiology and EHR systems would improve our rate of duplicate testing by seamlessly alerting the practitioner to the results of the recent study. However, our analysis represents a common means of EHR implementation, which is out-of-the-box installation. Integration of our systems involves cost and effort, which hopefully can be achieved shortly. At that time we plan to reanalyze our duplicate testing rate to ensure that the desired outcome is achieved.

## 5. Conclusion

Our study failed to demonstrate a reduction in the rate of duplicate transthoracic echocardiography testing after the implementation of an electronic health records system. We recognize several limitations of our analysis; nonetheless we feel that this data combined with other recent analyses should promote a more rigorous assessment of the initial claims of the benefits associated with EHR implementation. Transition to a computerized medical records system represents a significant and necessary advance in clinical medicine. However this transition has occurred on a timeline dictated by incentive payments and in many cases spearheaded by administrators as opposed to clinicians. The actual effects of health information technology on patient care and finances need to be closely examined as the systems are used under real-world conditions.

## Figures and Tables

**Table 1:** Baseline demographic characteristics and indications for the echocardiogram (LV function = left ventricular function, HTN HD = hypertensive heart disease).

Date	July 2007–June 2008	July 2009–June 2010	*p* value	July 2011–June 2012	*p* value
Total patients with TTEs	10399	7103		7317	

Age	73 ± 17	73 ± 17	NS	71 ± 17	0.01

Sex	Male 48%	Male 48%	NS	Male 48%	NS

Indications selected by ordering physician	(1) LV function (15%) (2) HTN HD (12%) (3) Dyspnea (11%)	(1) LV function (14%) (2) HTN HD (12%) (3) Dyspnea (11%)		(1) CHF (10%) (2) LV function (9%) (3) Dyspnea (7%)	NS
